# Letter to the Editor Rebuttal to Qi et al. (2025): “Alpha 9 integrin in spinal cord repair: a critical appraisal of mechanisms, circuitry, and translational potential”

**DOI:** 10.1186/s40478-025-02186-7

**Published:** 2025-11-26

**Authors:** Katerina Stepankova, Lucia Machova Urdzikova, Jessica C.F. Kwok, James Fawcett, Pavla Jendelova

**Affiliations:** 1 Institute of Experimental Medicine Czech Academy of Science, Videnska 1083, Prague 4, 14200 Prague, Czech Republic; 2 Faculty of Biological Sciences, University of Leeds, Leeds, LS2 9JT UK; 3 Department of Clinical Neurosciences, John Van Geest Centre for Brain Repair, University of Cambridge, Cambridge, CB2 0PY UK

## Abstract

In response to the commentary by Qi et al. (2025) on our recent study of α9 integrin in spinal cord repair, we provide clarifications on several factual and interpretative points. The α9 integrin binding site resides within the third fibronectin type III-like repeat of tenascin-C, which is not alternatively spliced, ensuring consistent ligand engagement. Multiple studies, including those by Dobbertin et al., Cimpean et al., and Tan et al. support the pro-regenerative role of α9 integrin and its ability to overcome CSPG-mediated inhibition through Src and FAK signalling. We further note that long-term studies are indeed warranted and have facilitated such research by depositing all constructs on Addgene. Contrary to the commentary’s claim, our study employed a spinal cord injury model, not a dorsal root crush, and histological data confirm the presence of a fibrotic scar. No evidence of neuropathic pain was observed at 12 weeks, indicating functional safety within the study timeframe. Together, these clarifications reaffirm that α9 integrin signalling directly promotes axon regeneration and that future work using temporally controlled systems will build on these findings.

To the Editor,

We read with interest the recent correspondence by Qi et al. (2025) entitled “Alpha 9 Integrin in Spinal Cord Repair: A Critical Appraisal of Mechanisms, Circuitry, and Translational Potential.” We appreciate their engagement with our work Stepankova et al. [1], but we wish to clarify several factual inaccuracies and to address specific points raised in their commentary. Regarding line 50 of Qi et al. (2025): Qi et al. raise uncertainty about the precise localization and splice regulation of the α9 integrin binding site on tenascin-C (TNC). We wish to clarify that the α9 integrin binding site is located within the third fibronectin type III-like (FNIII) repeat of tenascin-C, which is not subject to alternative splicing. This region contains the YVNGH motif, the canonical binding sequence for α9β1 integrin, and is conserved across vertebrate tenascin-C isoforms [2]. Thus, α9-mediated adhesion to tenascin-C does not depend on the inclusion or exclusion of alternatively spliced FNIII repeats.

Lines 54–57: Qi et al. question whether α9 integrin signalling promotes growth directly or whether it functions only by counteracting inhibitory cascades. Several independent studies, including our own publications (bolded below), have demonstrated that α9 integrin directly stimulates neurite outgrowth on tenascin-C substrates. Dobbertin et al. [3] showed that α9β1 engagement with tenascin-C promotes neuronal extension, and **Cimpean et al.** (Frontiers in Cellular Neuroscience) [4] further confirmed that tenascin-C supports axonal growth of α9 transduced DRGs. We also confirmed that activated α9 promotes axonal growth on inhibitory environment **Stepankova et al.** [5]. Conversely, **Tan et al.** [6] demonstrated that chondroitin sulphate proteoglycans (CSPGs) suppress integrin-mediated signalling through Src and FAK, providing mechanistic context for how α9 activation may overcome CSPG-driven inhibition. Finally, **Cheah et al.** [7] established that expression of either integrin or Kindlin alone produces only marginal regeneration, whereas co-activation of integrin with Kindlin is required for functional axon regrowth. Together, these studies substantiate our conclusion that α9 integrin activation acts through direct, pro-growth signalling rather than a secondary blockade of inhibition.

As such, we believe the comments by Qi et al. (2025) reflect a limited understanding of previous research and present a somewhat biased perspective. While we appreciate constructive feedback and acknowledge that there may be slight difference in clinical implications, disregarding the substantial contributions from fundamental research risks misguiding the field.

Lines 63–70: We agree that long-term functional studies are an important next step. To enable such investigations, all constructs used in our study have been deposited on Addgene, allowing other research groups to pursue these experiments. Future work should indeed examine neuropathic pain and autonomic dysreflexia as potential consequences of extensive regeneration. It is worth noting that in Stepankova et al. [1], animals were monitored for 12 weeks post-injury, and no behavioural or histological evidence of severe neuropathic pain was observed—such pathology would have been apparent by that timepoint if aberrant circuitry had formed.

Lines 73–80: Qi et al. incorrectly describe our experimental model as a dorsal root crush. In fact, the study employed a spinal cord injury (SCI) model, with clear histological evidence of a fibrotic scar, as shown in Figs. 2, 3 and 6 of publication Stepankova et al. [1]. The distinction is not trivial; the SCI model used reflects the complex glial and fibrotic environment of central lesions. However, to get closer to the clinical settings, experiments utilizing Horizon impactor contusion model are ongoing. So far, we can see regenerating axons in the lesion area. We agree that, should long-term expression of α9 integrin reveal any adverse effects, a temporal or inducible expression system would be a logical and prudent approach for future studies.

In conclusion, we thank Qi et al. for their thoughtful commentary but respectfully disagree with these misinterpretations. The α9 integrin binding site is not alternatively spliced, its growth-promoting role on tenascin-C is well-established, and our study indeed utilized a spinal cord injury model. We fully concur that longer-term, conditional studies are valuable next steps, and we have facilitated such research by making all constructs publicly available.

Sincerely,

Pavla Jendelova, on behalf of the authors.

Correspondence: pavla.jendelova@iem.cas.cz

## Data Availability

Our data will be made available on reasonable request.
